# Polymer-Based Shaping Strategy for Zeolite Templated Carbons (ZTC) and Their Metal Organic Framework (MOF) Composites for Improved Hydrogen Storage Properties

**DOI:** 10.3389/fchem.2019.00864

**Published:** 2019-12-17

**Authors:** Lerato Y. Molefe, Nicholas M. Musyoka, Jianwei Ren, Henrietta W. Langmi, Mkhulu Mathe, Patrick G. Ndungu

**Affiliations:** ^1^HySA Infrastructure Centre of Competence, Energy Centre, Council for Scientific and Industrial Research (CSIR), Pretoria, South Africa; ^2^Department of Chemical Sciences, University of Johannesburg, Johannesburg, South Africa; ^3^Department of Chemistry, University of Pretoria, Pretoria, South Africa

**Keywords:** hydrogen storage, physisorption, polymers of intrinsic microporosity, zeolite templated carbon, metal organic frameworks

## Abstract

Porous materials such as metal organic frameworks (MOFs), zeolite templated carbons (ZTC), and some porous polymers have endeared the research community for their attractiveness for hydrogen (H_2_) storage applications. This is due to their remarkable properties, which among others include high surface areas, high porosity, tunability, high thermal, and chemical stability. However, despite their extraordinary properties, their lack of processability due to their inherent powdery nature presents a constraining factor for their full potential for applications in hydrogen storage systems. Additionally, the poor thermal conductivity in some of these materials also contributes to the limitations for their use in this type of application. Therefore, there is a need to develop strategies for producing functional porous composites that are easy-to-handle and with enhanced heat transfer properties while still retaining their high hydrogen adsorption capacities. Herein, we present a simple shaping approach for ZTCs and their MOFs composite using a polymer of intrinsic microporosity (PIM-1). The intrinsic characteristics of the individual porous materials are transferred to the resulting composites leading to improved processability without adversely altering their porous nature. The surface area and hydrogen uptake capacity for the obtained shaped composites were found to be within the range of 1,054–2,433 m^2^g^−1^ and 1.22–1.87 H_2_ wt. %, respectively at 1 bar and 77 K. In summary, the synergistic performance of the obtained materials is comparative to their powder counterparts with additional complementing properties.

**Graphical Abstract:**
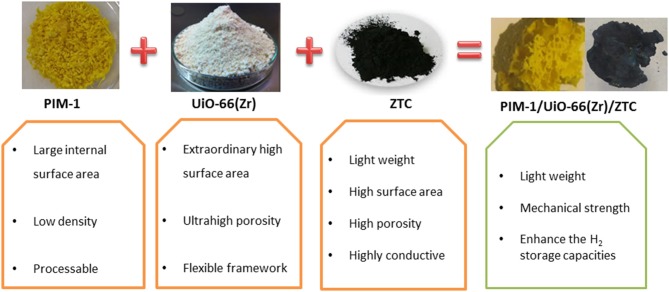
Preparation of PIM-1/UiO-66(Zr)/ZTC composite.

## Introduction

The current state-of-the-art for hydrogen (H_2_) storage in fuel cell vehicles is compression at 700 bar of which the storage tank is heavy, expensive and can also cause safety problems. Typically, type IV cylinder in fuel cell vehicles can store ≥5 wt. % of H_2_ at 700 bar (Hua et al., 2017). Therefore, strategies for developing alternative effective and efficient methods for storing H_2_ still remain a challenge. The proposed 2020 United States Department of Energy (DOE) targets for on-board H_2_ storage systems (include valves, pressure containment, cooling systems, etc.) are 4.5 wt. % and 0.030 kg H_2_/ L for gravimetric and volumetric storage, respectively (US Department of Energy, 2018). The tremendous research progress made in nanoporous materials for hydrogen storage applications shows great promise. Materials with high surface areas such as metal organic frameworks, carbons and porous polymers can store hydrogen through physisorption at safer low pressures and possess good reversibility as well as fast kinetics (Yang et al., 2011). Moreover, high gravimetric H_2_ capacities up to 8.9 wt. % at 77 K and 30 bar on activated carbons (Blankenship et al., 2017) and a maximum total of 10 wt. % on NOTT-112 MOFs can be achieved at 77 K and 77 bar (Yan et al., 2009). Whereas, at low pressures, maximum amounts of H_2_ adsorbed on MOFs are slightly lower and have been reported to be around 2.5 wt. % (Rowsell and Yaghi, 2006) while carbons such as CA-4700 have 3.9 wt % at 77 K and 1 bar (Blankenship et al., 2017). However, volumetric capacities remain low at same conditions (Kaye et al., 2007). Therefore, the properties of these adsorbents must be improved to achieve high gravimetric and volumetric capacities at both low and ambient temperatures to become practically viable for their inclusion into an on-board H_2_ storage system. Furthermore, nanoporous materials need modifications in order to enhance other physical properties such as thermal conductivity that is required for fast heat dissipation and mechanical strength required for better handling. These additional properties are crucial in practical applications.

Metal organic frameworks (MOFs) are a class of crystalline and highly porous hybrid materials consisting of metallic ions and organic ligands (Férey, 2008). The properties of MOFs can be tuned by using a variety of metal ions and organic linkers during synthesis (Hu and Zhao, 2015). Besides application in drug delivery, gas separation and catalysis, MOFs not only exhibit interesting properties for gas adsorption and storage but can also be used as sensors, among other applications (Murray et al., 2009; Della Rocca et al., 2011; Kreno et al., 2011; Stavila et al., 2014). In this study, the interest of zirconium-carboxylate Universitetet i Oslo (UiO-66(Zr)) MOF was due to its excellent mechanical, chemical, and thermal stabilities (Cavka et al., 2008). In addition to MOFs, conductive carbon nanostructured materials such as zeolite templated carbons (ZTCs) whose structure consists of a three-dimensional network of buckybowl-like nanographene (Nishihara et al., 2009) have emerged as attractive materials for H_2_ storage applications due to their high surface area, high porosity, light weight and high thermal conductivity (Yang et al., 2016).

The synthesis of porous composites has emerged as an attractive strategy for enhancing the intrinsic properties of the individual materials. For instance, Musyoka et al. (2017) investigated the *in-situ* compositing of the zirconium based UiO-66 MOF with reduced graphene oxide (rGO) and observed an increased surface area coupled with enhanced hydrogen uptake capacity for the rGO/Zr-MOF composite in comparison to pristine Zr-MOF. The mechanism of hydrogen adsorption in carbon material was reported to be through dissociation and chemisorption of hydrogen on the carbon sites with isolated hydrogen being easier to diffuse on the carbon materials (Wang et al., 2016). Particularly, composites of MOFs with carbon materials are of great interest because carbon materials have high thermal conductivity, which is beneficial for heat management during hydrogen cycling (Ngene et al., 2017). However, despite their enhanced hydrogen sorption behavior, MOF/Carbon composites still have processing challenges. Hence recently, fabrication of various types of polymer based carbon and MOF composites have been investigated and identified as an interesting strategy not only for improving the performance but also for shaping of these materials.

On the other hand, a highly processable polymer of intrinsic microporosity (PIM-1) is an attractive adsorbent material that is soluble in common solvents and it can be casted into mechanically stable structures (Budd et al., 2004). However, most focus has been on improving the adsorptive properties of these materials for high uptake capacities and very little effort has been done on tailoring the materials for large scale applications properties. In a recent study by Tien-Binh et al. (2018) defect-free mixed matrix membranes (MMM) made up of PIM-1 and UiO-66-NH_2_ filler were prepared by *in-situ* chemical cross-linking of UiO-66-NH_2_ with PIM-1 during polymer synthesis to improve the poor polymer-filler adhesion. It was reported that the *in-situ* chemical reaction between 1,4-dicyanotetrafluorobenzene monomer and amine groups of UiO-66(Zr) MOF lead to direct grafting of PIM-1 onto the MOF surface and thus enhancing polymer-filler adhesion and gas separation performance (higher permeability and selectivity for all the gases tested).

There have been other several studies on PIM-1/UiO-66 MMM (Khdhayyer et al., 2017; Tien-Binh et al., 2018; Yu et al., 2019), however their scope was limited to membranes for gas separation applications. Several other studies have also reported on other types of MOF/Carbon composites such as MOF-5/expanded natural graphite (ENG) (Liu et al., 2012), MIL-101(Cr)/ZTC (Musyoka et al., 2016) and HKUST-1/graphene platelets (Hassan et al., 2019). Additionally, other MOF/polymer composites such as PIM-1/MIL-101 (Cr) (Khdhayyer et al., 2019), PIM-1/UiO-66 (Khdhayyer et al., 2017), PIM-1/UiO-66-NH_2_ (Tien-Binh et al., 2018), PIM-1/UiO-66-CN (Yu et al., 2019) and Matrimid/UiO-66 MMMs (Marti et al., 2018) have been reported. However, no work has been done on composites materials consisting of the three pristine materials (carbon/polymer/MOF) for H_2_ adsorption applications. In our previous work (Molefe et al., 2019) we reported that upon increasing the MOF loading in PIM-1/MIL-101(Cr) composites, the surface area, pore volume and H_2_ adsorption capacity increased significantly. Our findings showed that 80 wt% loading of MIL-101(Cr) onto PIM-1 exhibits enhanced H_2_ uptake with no pore blocking effects. Hence, the optimized filler loading of 80 wt% was also chosen for the current study. However, the polymer/MOF composites still lacked thermal conductivity. Therefore, it is on this basis that the current study presents the merging of physico-chemical properties of ZTC, PIM-1 and UiO-66(Zr) MOF into mouldable functional composites. In as much as all the three pristine materials can serve as individual H_2_ adsorbents, ZTC in this case serves as the thermal conductivity enhancer whereas the PIM-1 also serves as the binder material.

## Materials and Methods

### Chemicals

In this study, the following chemicals were used: Potassium carbonate (K_2_CO_3_, Sigma-Aldrich, 99+%), N,N-dimethylformamide (DMF, Sigma-Aldrich, 99.8%), 5,5′,6,6′-tetrahydroxy-3,3,3′,3′-tetramethyl-1,1′-spirobisindane (TTSBI, Sigma-Aldrich), 1,2,2-tetrachloroethane (TCE, Sigma-Aldrich, 98.0+%), 2,3,5,6 tertrafluoroterephthalonitrile (TFTPN, Sigma-Aldrich, 99%), 13X powder (Sigma-Aldrich, ~2 μm average particle size), furfuryl alcohol (FA, Sigma-Aldrich, 97+%), hydrofluoric acid (HF, Ace chemicals, 97+%), hydrochloric acid (HCl, Ace chemicals, 37+%), Anhydrous chloroform (CHCl_3_, Sigma-Aldrich, 99.9+%), Zirconium chloride (ZrCl_4_, Sigma-Aldrich, 99.5+%), terephthalic acid (BDC, Sigma-Aldrich, 98%), formic acid (HCOOH, Sigma-Aldrich, 99.5+%), methanol (CH_3_OH, Sigma-Aldrich, 99.9+%), ethanol (Ace chemicals, 90%) and de-ionized water were purchased and used without further purification. Hydrogen and nitrogen (N_2_) gases of ultra-high purity grade purchased from Afrox Company, South Africa were used for sample analysis.

### Experimental Procedure

#### Synthesis of ZTC

In this study, the synthesis of ZTC was prepared based on the previously reported two step procedure involving furfuryl alcohol impregnation of zeolite 13X followed by its chemical vapor deposition (Masika and Mokaya, 2013; Musyoka et al., 2016). In summary, zeolite 13X (24 g) was dried in the vacuum oven at 120°C under vacuum for 12 h prior to its impregnation with 80 ml of furfuryl alcohol (FA) at room temperature. After subjecting the FA/zeolite mixture to magnetic stirring for 24 h, the mixture was filtered and washed with a few drops of ethanol to remove the excess furfuryl alcohol. Subsequently, the FA /zeolite mixture was placed on high purity quartz crucible and transferred into an alumina ceramic tube furnace where it was polymerised at 80°C for 24 h under argon gas flow (100 mL min^−1^). Thereafter, polymerisation was continued by heating the mixture at 150°C for further 8 h. The resulting hybrid was further heated up to 700°C under argon flow to allow stabilization of the composite. After 3 h, still at 700 °C, the gas flow was switched to a mixture of ethylene/argon (10% ethylene in argon) and held for 3 h. The gas flow was switched back to argon only, while the temperature was being ramped up to 900°C and carbonization occurred for 3 h. Still under argon flow, the resulting product, referred to as zeolite/carbon composite was then cooled down to room temperature. The sample was washed in 10% aqueous solution of hydrofluoric acid for 24 h, followed by diluting the aqueous solution to 2 L with deionised water and filtered. The sample was further refluxed in 10% aqueous solution of hydrochloric acid for 24 h. Finally, the resulting carbonaceous materials called ZTC was collected by filtration and washed with 2L of deionised water and dried in a vacuum oven at 120°C for 12 h.

#### Synthesis of UiO-66(Zr)

The synthesis method for UiO-66(Zr) was as reported in a previous study (Ren et al., 2014) with some changes. In a typical procedure, 1.06 g of ZrCl_4_ and 0.68 g of BDC were dissolved in 50 mL of DMF and sonicated for 30 min. The resulting mixture along with 17.13 mL of formic acid was transferred into a 250 mL round bottom flask connected to a reflux system. Subsequently, the system was placed in an oil bath pre-heated at 120°C and kept for 4 h. Finally, the obtained white precipitate was collected by centrifugation followed by washing with ethanol at 60°C. The final product was then dried at 90°C in a conventional oven.

#### Synthesis of PIM-1

PIM-1 was prepared through a double nucleophilic aromatic substitution reaction between two monomers (TTSBI and TFTPN) in the presence of anhydrous K_2_CO_3_ catalyst as per typical procedure reported by Budd et al. (2004). In this case, a mixture of equimolar ratio amounts of TTSBI and TFTPN and certain amount of anhydrous K_2_CO_3_ catalyst (8 equivalent with respect to monomers) was evacuated and backfilled with nitrogen gas prior to dissolving in 100 ml of anhydrous DMF. The mixture was kept under nitrogen flow and heated to 65°C under vigorous magnetic stirring. After 72 h, the resulting yellow precipitate was cooled and dispersed in 300 ml of deionised water and stirring continued for 1 h. The obtained solid was collected by vacuum filtration followed by dissolving in chloroform and re-precipitated in methanol. The final product was dried at 80°C for 12 h in a vacuum oven.

#### Synthesis of PIM-1/UiO-66(Zr)/ZTC Composites

Different composites samples of PIM-1/80 wt% UiO-66(Zr), PIM-1/80 wt% ZTC and PIM-1/UiO-66(Zr)/ZTC (80 wt%) were prepared by physical mixing of pre-synthesized polymer and MOF/carbon powders using TCE solvent. The UiO-66(Zr) and ZTC powders were degassed at 200°C for 4 h prior to experiment to remove any adsorbed solvent or water. In the case of PIM-1/80 wt% UiO-66(Zr), a procedure similar to the recently reported method for PIM-1/80 wt% MIL-101(Cr) composite (Molefe et al., 2019) was followed but using a different type of MOF. By modifying the synthetic method for PIM-1/80 wt% UiO-66(Zr) through substitution of UiO-66(Zr) with ZTC, a black monolith with 80 wt% of ZTC and 20 wt% PIM-1 was obtained. On the other hand, the PIM-1/UiO-66(Zr)/ZTC (80 wt%) composite consisting of 20 wt% PIM-1, 40 wt% ZTC and 40 wt% UiO-66(Zr) in TCE solvent was prepared through the same solvent impregnation method. The resulting mixture was then heated at 150°C to remove the residual solvent inside the monolith composite.

### Sample Characterization

The crystallinity analyses of the samples was conducted using the powder X-ray diffractometer (PANalytical X'Pert Pro), with a Pixcel detector (Cu-K_α_ radiation λ = 0.154 nm). The powder X-ray diffraction (PXRD) patterns were measured at scanning rate of 2° min^−1^ at room temperature. TGA/SDTA 851^e^ instrument (Mettler, Toledo) was used for thermogravimetric analysis (TGA). The samples were heated at a heating rate of 10°C min^−1^ from 46 to 1,000°C. The analyses were carried out under air (60 mL min^−1^) and nitrogen flow (40 mL min^−1^) as a balance gas. All Fourier transform infra-red (FTIR) spectra were obtained using a Perkin-Elmer Spectrum 100 FTIR spectrometer, working in the range from 4,000 to 400 cm^−1^ with a resolution of 4 cm^−1^ in attenuated total reflection (ATR) mode. Prior to each FTIR measurements, 8 scans of the background were collected. The morphology of the obtained samples was observed using the focused ion beam scanning electron microscope (Carl Zeiss Auriga Cobra). The nitrogen and hydrogen sorption (up to 1 bar) isotherms were obtained from a Micrometrics ASAP 2020 HD analyser at 77 K. The specific surface areas by Brunauer-Emmett-Teller (BET) theory was obtained from the nitrogen physisorption isotherms. Prior to the gas sorption experiment, the samples were degassed in the degassing port under vacuum (down to 10^−7^ bar) at 200°C for at least 8 h for removal of moisture or other volatile residues.

## Results and Discussion

**Figure 1:**
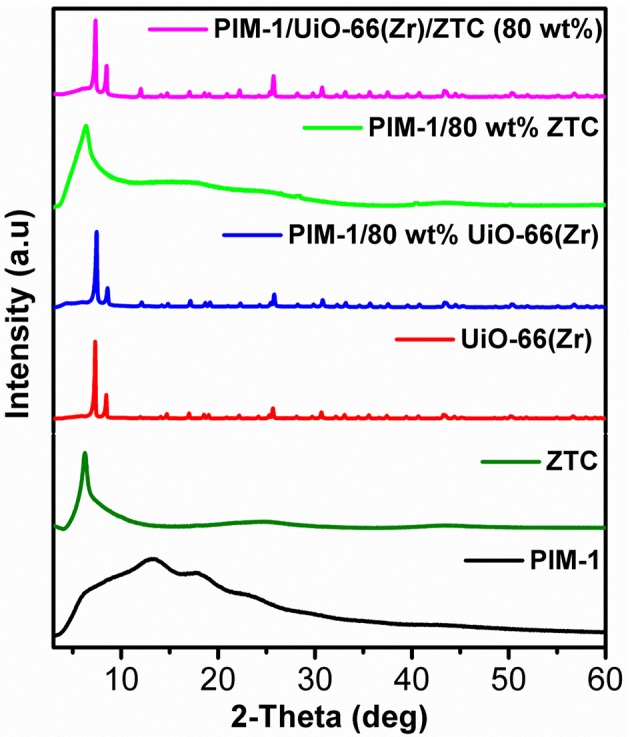
Stacked PXRD patterns of PIM-1, ZTC, UiO-66 (Zr) and their corresponding composites samples.

The PXRD pattern for the prepared UiO-66 (Zr) exhibited the main characteristic peaks which are consistent with the previous reported studies (Ren et al., 2014) and confirms the formation of pure crystalline MOF. The observed crystallinity complement the respective SEM image (Figure 4d), showing highly defined octahedral shaped crystals with sharp edges as an attribute of the presence of 100 equivalent of modulator (formic acid) used during their synthesis. The ZTC sample showed a diffraction peak at 6.2° 2-theta similar to that of the zeolite 13X template, indicating a replication of zeolite 13X-type structural pore ordering in ZTC and also confirms successful templating process (Xia et al., 2011; Musyoka et al., 2018). After incorporating the UiO-66 (Zr) and ZTC into the PIM-1 matrix to obtain PIM-1/80 wt%UiO-66(Zr) and PIM-1/80 wt% ZTC composites materials, the characteristics peaks of fillers were still present and dominant. Thus, indicating that the crystal structures of fillers were retained upon the crosslinking process between the polymer matrix and filler materials. As observed in Figure 1, the diffraction patterns of composite materials are dominated by strong diffraction peaks resulting from UiO-66(Zr) and ZTC, respectively, coupled with a bit of amorphousness from PIM-1's presence which is not that much prominent because of its low weight percentages (20 wt%). On the other hand, for PIM-1/UiO-66(Zr)/ZTC (80 wt%), the characteristic peaks of UiO-66(Zr) are dominant and clearly observed compared to those of ZTC and amorphous nature of PIM-1 and the crystal phase structure of UiO-66(Zr) is still maintained.

**Figure 2:**
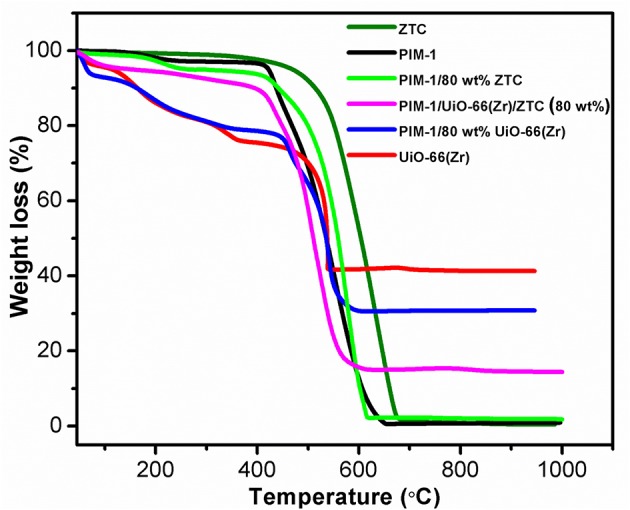
TGA thermograms of PIM-1, UiO-66 (Zr), ZTC and their corresponding composites samples.

To further investigate the properties of the obtained composites, thermal behavior of all the pristine and composites materials was investigated. As depicted in the TGA curves (Figure 2), the thermal stability was found to follow the order of ZTC>PIM-1/80 wt% ZTC> UiO-66(Zr)> PIM-1/80 wt% UiO-66(Zr)> PIM-1>PIM-1/UiO-66(Zr)/ZTC (80 wt%). Even though the incorporation of ZTC led to the improvement of thermal stability of PIM/80 wt% ZTC composite, it was interesting to note that the inclusion of UiO-66(Zr) in the composite containing the 3 filler materials (PIM-1/UiO-66(Zr)/ZTC (80 wt%)) had lesser thermal stability but still within the attractive ranges for most porous materials. In general, the thermal stability of PIM-1 (up to 415 °C) is known to be due to the presence of strong interactions of the nitrile groups (Du et al., 2008). On the other hand, UiO-66(Zr) weight loss at temperatures below 150 °C is often ascribed to the evaporation of adsorbed solvent and/or water molecules. The additional weight loss appearing in the range of 150 to 362 °C is attributed to the dehydroxylation of Zr_6_ clusters before the commencement of the final weight loss starting from 475 °C until the MOFs finally degrades to ZrO_2_. This trend in weight loss is observed in all the MOF-containing composites synthesized in this study.

**Figure 3:**
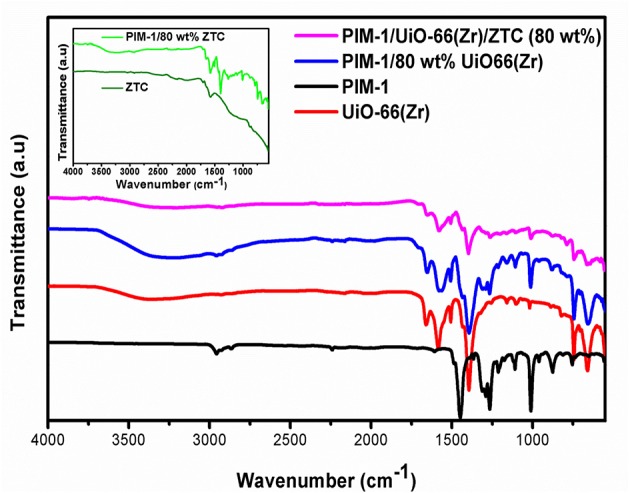
FTIR spectra of UiO-66(Zr), PIM-1/80 wt% UiO-66(Zr), PIM-1/UiO-66(Zr)/ZTC (80 wt%) in comparison with pristine PIM-1 and an insert showing the ZTC and PIM-1/80 wt% ZTC spectra.

Fourier transform infra-red was used for probing and confirming the chemical composition of the resulting composites and the cross-linking between the PIM-1 matrix and the filler materials (ZTC and UiO-66(Zr)). In Figure 3, the FTIR spectrum of pristine PIM-1 displays the C-O stretch (1,213–1,250 cm^−1^) (Chaukura and Maynard-Atem, 2015), and the characteristic nitrile (–C≡N) stretch (around 2,229 cm^−1^) (Patel and Yavuz, 2012). The intense peak around 1,446 cm^−1^ is due to C = C stretch vibrations and the other peaks observed at 1,105–1,015 cm^−1^ and 872 cm^−1^ can be assigned to C-C and C-O stretching vibrations, respectively. On the other hand, the IR spectrum of pristine UiO-66(Zr) showed characteristic vibration peaks of benzene ring at 1,510 and 1,403 cm^−1^ which agrees with reported literature (Luan et al., 2015). The intense doublet peaks at 1,657 and 1,581 cm^−1^ could be attributed to the in- and out-of-phases stretching modes of the carboxylate groups that are present in terephthalic acid linker (Zhu et al., 2014). The peaks at around 811, 748 and 658 cm^−1^ corresponds to the O-H and C-H vibrations in the BDC ligand (Yang et al., 2017).

A strong broad peak observed in the range of 3,300–3,500 cm^−1^ is attributed to the presence of hydroxyl groups from moisture adsorbed onto the surface of UiO-66 and composite materials. According to Nishihara et al. (2009), the idealized molecular structure of ZTC has edge sites of the buckybowl units containing different types of functional groups and a significant amount of oxygen. In this case, spectra of the pristine ZTC and PIM-1/80 wt% ZTC composite (Figure 3) showed a peak at around 1,720 cm^−1^ which can be ascribed to C = O stretching and a distinct band at 1,600 cm^−1^ assigned to the presence of carbonyl groups. The weak broad band in the ranges of 1,000–1,400 cm^−1^ for the ZTC sample was also observed by Nishihara et al. (2009) and can be attributed to C-O stretch. At the high carbonization temperature of 900°C, during chemical vapor decomposition process, phenolic OH groups are completely decomposed and hence the absence of the typical broad band in the range of 3,200–3,400 cm^−1^ in the pristine ZTC (Fukuhara et al., 2013). On the other hand, the effect of PIM-1 on the composites (PIM-1/80 wt% UiO-66(Zr) and PIM-1/UiO-66(Zr)/ZTC (80 wt%)) was found to be minimal and the expected PIM-1 peaks were masked by the prominence of UiO-66(Zr) peaks. However, in PIM-1/80 wt% ZTC composite all of the PIM-1 peaks were clearly observed. Due to the absence of new peaks in the resulting PIM-1/MOF/ZTC composite, it was inconclusive to confirm whether there was cross-linking that occurred between the polymeric binder and filler materials.

**Figure 4:**
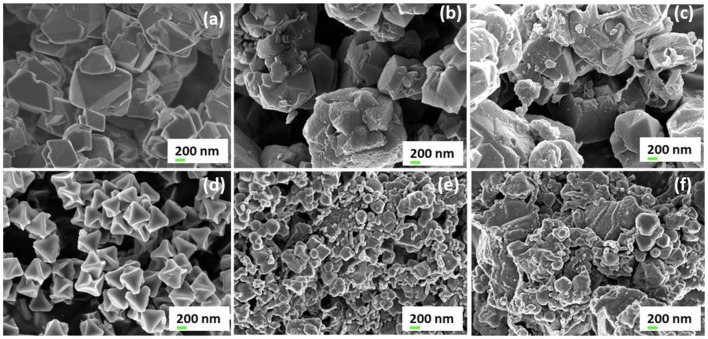
High resolution SEM images (mag. 20000×) of **(a)** zeolite 13X, **(b)** ZTC, **(c)** PIM-1/80 wt% ZTC, **(d)** UiO-66(Zr), **(e)** PIM-1/80 wt% UiO-66(Zr), and **(f)** PIM-1/UiO-66(Zr)/ZTC (80wt%).

The SEM images of both parent zeolite and resulting ZTC neat materials (as seen in Figures 4a,b,c) clearly show almost similar octahedral pyramidal morphology as earlier reported by Musyoka et al. (2016) and Yang et al. (2007). Octahedral shaped crystal morphology for the UiO-66(Zr) was also observed and is consistent with the previous reports (Ren et al., 2014). On the other hand, SEM images for composites materials confirm that the ZTC and UiO-66(Zr) are well-dispersed and embedded in PIM-1 matrix phase. In Figure 4f, the dominating MOF and ZTC particles are seen closely attached together by PIM-1, indicating a good distribution of filler materials on the matrix as earlier reported (Khdhayyer et al., 2017). Furthermore, the SEM images of the three composites do not show a sieve-in-a-cage morphology which is a characteristic for poor surface adhesion of filler particles to PIM-1. Thus, dissolving PIM-1 in TCE solvent lead to fluoride chain-ends of PIM-1 to interact with the hydroxyl functional groups of the BDC linker on the surface of UiO-66(Zr) MOF and prevent the polymer from blocking the MOF pores (Tien-Binh et al., 2016).

**Figure 5:**
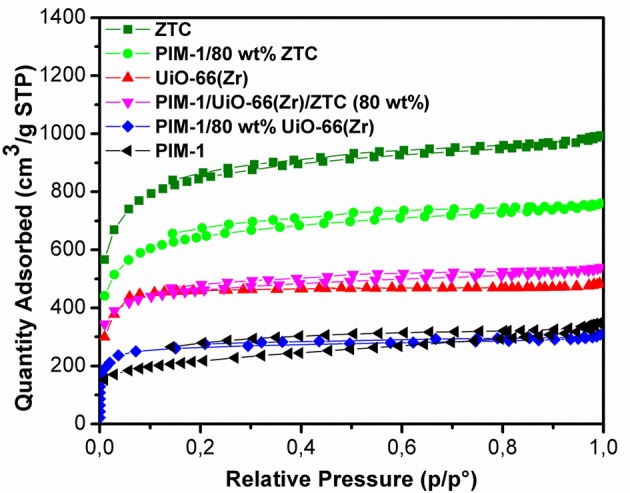
N_2_ sorption isotherms of pristine and composites materials at 77 K.

Figure 5 presents the N_2_ adsorption-desorption isotherms of the pristine PIM-1, powder fillers and the resulting composites materials measured at 77 K. The nitrogen sorption isotherms of ZTC and UiO-66 (Zr) show a steep/linear adsorption rise at low pressure ranges (0.0–0.1), indicating their microporous nature. The same type I isotherm for microporous characteristic was maintained after the inclusion of PIM-1 and this is in agreement with SEM and PXRD results, wherein neither the morphology nor the crystal structure of MOF was destroyed. PIM-1 and PIM-1/80 wt% UiO-66(Zr) exhibited a type I isotherm coupled with type IV isotherm behavior, indicating the presence of both micropores and mesopores. The surface area of PIM-1 increased significantly from 785 to 1,767 m^2^g^−1^ and to 2,433 m^2^g^−1^ upon addition of 40 wt% UiO-66 (Zr)/40 wt% ZTC mixture and 80 wt% ZTC fillers respectively, which is equivalent to 3-fold increase as shown in Table 1. On the contrary, PIM-1/80 wt% UiO-66(Zr) exhibited a slight increase which is <2-fold increase. In most studies, MOF-polymer composites can only achieve about 60% of the expected BET values due to pronounced pore blocking effects as was reported by other researchers such as MIL-101(Cr)@NIPAM (Wickenheisser et al., 2016), HKUST@HIPE (Schwab et al., 2008) and UiO-66@polyurethane (Pinto et al., 2013). Whereas, our composites have reached over 80% of the estimated surface areas with an exception of PIM-1/80 wt% UiO-66(Zr) sample which achieved 62.8% suggesting that there are reduced pore blocking effects in ZTC containing samples. This can be attributed to ZTC's high level of stability toward PIM-1 disturbance, whereby it was seen that the ZTC-based composites retained most of its physical properties as compared to PIM-1/80 wt% UiO-66(Zr). Similar observations were reported in a recent study for PIM-1/MIL-101 MOF and PIM-1/AX21 activated carbon (Tian et al., 2019), wherein the activated carbon based composites materials showed enhanced physical properties and best H_2_ storage properties at both 0.1 and 10 MPa adsorption measurements.

**Table 1:** Summary of the physical properties of pure materials and their respective composites.

**Sample**	**Measured BET SSA (m^**2**^g^**−1**^)^a^**	**Estimated BET SSA (m^**2**^g^**−1**^)^b^**	**Micropore area (m^**2**^g^**−1**^)^c^**	**Total pore vol. (cm^**3**^g^**−1**^)^d^**	**Measured H_**2**_ uptake (wt. %)^e^**	**Estimated H_**2**_ uptake (wt. %)^f^**
Pristine ZTC	3,206	-	3,004	1.54	2.38	-
PIM-1/80 wt% ZTC	2,433	2,722	2,262	1.18	1.87	2.11
PIM-1/UiO-66(Zr)/ZTC (80 wt%)	1,767	2,200	1,668	0.83	1.65	1.70
Pristine UiO-66(Zr)	1,903	-	1,882	0.75	1.36	-
PIM-1/80 wt% UiO-66(Zr)	1,054	1,679	1,014	0.48	1.22	1.29
Pristine PIM-1	785	-	707	0.54	1.02	-

a *BET surface area measured from N_2_ adsorption isotherms at 77 K*.

b *BET surface area calculated as the sum of the mass-weighted surface areas of the MOF/ZTC (fillers) and PIM-1 (matrix) from this formula: $BET\;\left(estimated\right)=\frac{wt\%\;of\;filler}{100}\times BET_{filler}\;m^{2}g^{-1}+\;\frac{wt\%\;of\;matrix}{100}\times BET_{matrix}\;m^{2}g^{-1}\;$*.

c *From t-plot*.

d *Total pore volume determined from H-K analysis by uptake at p/p ~0.99*.

e *Hydrogen adsorbed at 77 K and 1 bar*.

f *The estimated H_2_ uptake calculated as the sum of the mass-weighted H_2_ adsorption capacity of the MOF/ZTC (fillers) and PIM-1 (matrix) from this formula: $H_{2}\;uptake\;\left(estimated\right)=\frac{wt\%\;of\;filler}{100}\times {H_{2}\;uptake}_{filler}\;wt.\%+\;\frac{wt\%\;of\;matrix}{100}\times {H_{2}\;uptake}_{matrix}\;wt.\%$*.

**Figure 6:**
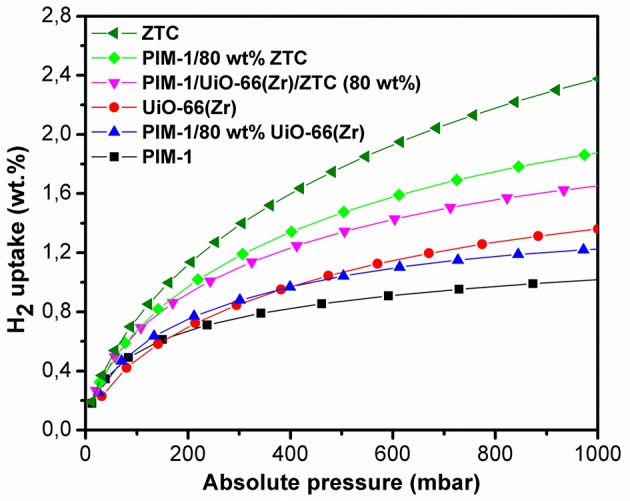
H_2_ adsorption isotherms of pristine PIM-1, UiO-66 (Zr), ZTC and their corresponding composites materials at 77 K and 1 bar.

The hydrogen adsorption curves of PIM-1/80 wt% ZTC, PIM-1/UiO-66(Zr)/ZTC (80 wt%) and PIM-1/80 wt% UiO-66(Zr) presented in Figure 6 showed a slight decrease ( ≤ 11% loss) in H_2_ uptake which is still at acceptable high capacity values (1.87, 1.65, and 1.22 wt. %, respectively) and comparable to the estimated values (2.11, 1.70, and 1.29 wt. %, respectively). The relatively high H_2_ uptake can be attributed to the resulting high surface area and microporous network resulting when micropores of PIM-1 interconnect with the inner pores of UiO-66 (Zr) and ZTC forming an inter-connected micropore network.

The studies shown in Table 2 are not the only ones where PIM-1 was considered as polymer matrix in carbon and MOFs composites. A wide range of PIM-1 based composites have been reported for other applications, mostly in MMMs for gas separation application. Some examples include a combination of PIM-1 with fillers such as GO (Alberto et al., 2017), carbon nanotubes (Koschine et al., 2015), MOF-74 (Tien-Binh et al., 2016), ZIF-67 (Wu et al., 2018), ZIF-8 (Benzaqui et al., 2016), and COFs (Wu et al., 2017) MMMs. It is only recently, that there seems to be a growing interest of PIM-1 composites fabrication toward H_2_ storage applications. Our MOF composites exhibited H_2_ uptake values which are within the same range with others reported in the literature. Table 2 demonstrates that the H_2_ uptake for all composites materials increase with the loading amount of fillers and it correlates well with the BET surface area of all samples. Another observation common in all composites, is that their H_2_ uptake capacities are equal to the pristine materials or in some cases (PIM-1/MIL-101(Cr) (80 wt%) (Molefe et al., 2019) and PIM-1/PAF (37.5 wt%) (Rochat et al., 2017) found to exceed the expected values which signify good retention of intrinsic properties of the fillers. Nonetheless, just like many other previously reported materials, our composites are also yet to meet all the United States Department of Energy (DOE) 2020 targets set for on-board automobile hydrogen storage systems (Lim et al., 2010).

**Table 2:** Comparison of N_2_ and H_2_ sorption data of PIM-1 based composites materials from literature.

**Composites**	**Measure BET surface area (m^**2**^g^**−1**^)**	**Experimental H_**2**_ uptake (wt. %) at 77 K and 1 bar**.	**References**
PIM-1/MIL-101(Cr) (80 wt%)-Monoliths	2,333	1.73	(Molefe et al., 2019)
PIM-1/AX21 (60 wt%)	2,075	1.90	(Tian et al., 2019)
PIM-1/MIL-101 (40 wt%)-Films	1,580	1.11	(Tian et al., 2019)
PIM-1/PAF-1 (37.5 wt%)	1,639	1.15	(Rochat et al., 2017)
PIM-1/80 wt% UiO-66(Zr)	1,054	1.22	This work
PIM-1/80 wt% ZTC	2,433	1.87	This work
PIM-1/UiO-66(Zr)/ZTC (80 wt%)	1,767	1.65	This work

## Conclusion

In summary, the PIM-1 based composites consisting of UiO-66(Zr), ZTC, and UiO-66(Zr)/ZTC mixture were successfully fabricated. Our results showed that after compositing, the obtained monoliths still maintained the meso-microporosity and other favorable H_2_ uptake properties. All the composites materials showed better hydrogen storage performances which are in agreement with the predicted values with negligible loss of < 12%. The results also suggest that the ZTC-based composites materials (BET surface areas 1,767–2,433 m^2^g^−1^) were more stable and managed to retain most of the pristine ZTC intrinsic properties, with less pore blocking effects when compared to the MOF based materials (BET surface area 1,054 m^2^g^−1^). It was found that the interactions between UiO-66(Zr) and PIM-1 matrix were more physically than chemical since no bond formation was observed. This study presented an easy-to-fabricate PIM-1/80 wt% ZTC, PIM-1/UiO-66(Zr)/ZTC (80 wt%) composites that had not been reported before. However, these composites materials still need further modifications to improve their H_2_ uptake capacities as gravimetric capacities significantly >4.5 wt. % are required to achieve the set DOE targets, of which is possible to achieve at pressures slightly higher than 1 bar. Future work will focus on thermal conductivity measurements of the composites and compare that to other closely similar materials.

## Data Availability Statement

The datasets generated for this study are available on request to the corresponding author.

## Author Contributions

LM was responsible for synthesis, characterization of samples, and drafting the manuscript. NM and PN contributed to the experiment design and interpretation of data. JR, HL, and MM have also contributed to the interpretation of results and discussion.

### Conflict of Interest

The authors declare that the research was conducted in the absence of any commercial or financial relationships that could be construed as a potential conflict of interest.
